# Implementation of the healthy heart tool- an algorithm with potential cardiometabolic health benefits in persons with severe mental illness

**DOI:** 10.1186/s12888-025-06578-w

**Published:** 2025-02-25

**Authors:** Elisabeth Haug Lund-Stenvold, Petter A. Ringen, Ole A. Andreassen, Torfinn L. Gaarden, Cecilie B. Hartberg, Erik Johnsen, Silje Myklatun, Kåre Osnes, Kjetil Sørensen, Arne Vaaler, Serena Tonstad, John A. Engh, Anne Høye

**Affiliations:** 1 Department of Clinical Medicine, UiT The Arctic University of Norway, Tromsø, Norway; 2 Division of Mental Health and Substance Abuse, University Hospital of North Norway, Tromsø, Norway; 3 Division of Mental Health and Addiction, Oslo University Hospital, Oslo, Norway; 4 Institute of Clinical Medicine, University of Oslo, Oslo, Norway; 5 Division of Mental Health and Addiction, NORMENT Centre of Excellence, Oslo University Hospital, Oslo, Norway; 6 Division of Mental Health and Substance Abuse, Diakonhjemmet Hospital, Oslo, Norway; 7 Division of Psychiatry, Haukeland University Hospital, Bergen, Norway; 8 Department of Clinical Medicine, University of Bergen, Bergen, Norway; 9 Division of Mental Health, St Olav’s University Hospital, Østmarka, Trondheim, Norway; 10 Department of Acute Psychiatry, St. Olav’s University Hospital, Trondheim, Norway; 11 Department of Mental Health, NTNU, Trondheim, Norway; 12 Section for Preventive Cardiology Endocrinology, Morbid Obesity and Preventive Medicine, Oslo University Hospital, Oslo, Norway; 13 Division of Mental Health and Addiction, Vestfold Hospital Trust, Tønsberg, Norway

**Keywords:** Severe mental illness, Cardiovascular risk, Mortality risk reduction

## Abstract

**Background:**

Cardiometabolic diseases are the main causes of death in persons with severe mental illness (SMI), highlighting the need to improve management of cardiovascular risk factors in both primary and specialized health care. The “Healthy Heart Tool” aims at helping health care workers to identify persons at risk, and to initiate proper interventions. Here we investigate if the recommendations in the Healthy Heart Tool are followed one year after implementation and whether implementation of the tool improved cardiometabolic risk factors in SMI.

**Methods:**

Data from 270 individuals with SMI from six Norwegian hospitals were collected at baseline and at 12 months after implementation of the Healthy Heart Tool throughout the health care services. Changes from baseline to 12 months follow-up were analyzed using chi-square and independent t-tests, whereas implementation effects were analyzed using logistic general linear mixed models.

**Results:**

After implementing the Healthy Heart Tool, significantly more persons received dietary advice and/or salt restriction advice (75.5% vs. 84.8%, *p* = 0.035). After controlling for Body Mass Index (BMI) ≥ 30 and sex, there was an odds ratio (OR) of 8.9 (95% CI 1.42–55.77) for receiving dietary advice and/or advice on salt reduction. There was a significant reduction (*p* = 0.016) in numbers of participants with high levels of total serum cholesterol ≥ 5 mmol/ (54.4% vs. 46.3%).

**Conclusions:**

Implementing the Healthy Heart Tool can increase awareness of cardiovascular risk factors in patients with SMI. The intervention increased the proportion of individuals who received dietary and salt reduction advice and decreased the proportion of individuals with high cholesterol levels. However, due to the small numbers, these results should be interpreted with caution. Nonetheless, the findings suggest that the Healthy Heart Tool may be an effective means for improving the management of cardiovascular risk factors in individuals with SMI in typical clinical settings.

**Trial registrations:**

The trial was retrospectively registered in ClinicalTrials.gov 29.01.25, ID NCT 06807242.

## Background

Persons with severe mental illness (SMI), in the current study referring to people with schizophrenia, bipolar disorder or severe depression, have increased mortality risk compared to the general population [1–3]. Several lines of evidence suggest that the primary cause of the higher mortality in persons with SMI is inadequate disease prevention and treatment, in particular of cardiovascular disease (CVD) [1, 4, 5].

Persons with SMI have 53% higher risk of CVD [6], and 85% higher risk of dying from CVD, compared to the general population [6]. In addition to CVD, respiratory and metabolic diseases are the main contributors to reduced life expectancy in persons with SMI, followed by cancer and infectious diseases [7–11]. Moreover, in this group, as in the general population, sedentary behavior and smoking are independently associated with increased risk of CVD and type 2 diabetes [12, 13]. However, persons with SMI are more likely to smoke cigarettes [14], the amount of nicotine use per day is higher [15], they are more nicotine dependent [16] and they are less likely to receive smoking cessation advice than the general population [17–20]. People with SMI are less likely to meet physical activity guidelines and they are significantly more sedentary than age and gender matched controls [13]. They are also shown to have poorer dietary patterns with higher total energy and sodium intake, less vegetables and fruit and a higher intake of takeaway and other convenience food compared to their counterparts without SMI [21–23].

International guidelines recommend a structured estimation of the total CVD risk on an individual level as a measure to obtain the best primary intervention [24, 25]. In the general population the combination of interventions using physician reminders, dedicated personnel to deliver screening and provision of financial incentives has proved to be effective in screening for CVD risk factors [26]. The combination of unhealthy lifestyle, underlying physical disease and the potential metabolic side effects of antipsychotic medications calls for early intervention and screening for modifiable risk factors in persons with SMI, both in primary and specialist health care [27].

Despite these recommendations, persons with SMI in primary care are screened for CVD risk at a significantly lower rate than persons with diabetes [28–30], and screening rates for any CVD in people with SMI are significantly lower than in the general population [31, 32]. The prescription rates for antiplatelet, anticoagulant and lipid lowering agents are lower for persons with schizophrenia than in the general population [31, 32]. The NICE guidelines for treatment of individuals with psychosis and schizophrenia from 2014 recommend annual physical exams for people with SMI [33]. Other guidelines recommend routine monitoring of CVD and type 2 diabetes risk at given intervals in persons with SMI [34].

Despite the well documented CVD comorbidity and shortened life span in SMI, it has been difficult to reduce the CVD risk in clinical practice [35]. Individual lifestyle coaching or coordination of care of cardiometabolic risk factors in persons with abdominal obesity and schizophrenia spectrum disorder have, unfortunately, shown little effect [36], underlining the need for structural approaches. Patient factors such as psychiatric symptom load, reduced ability to interpret physical symptoms and reduced self-care ability are important, but other possible reasons might be associated with practical real-world issues such as health care logistics, lack of communication between specialists, fragmented multilevel services (primary care and hospitals), as well as stigma issues and a lack of sufficient training in health care workers to recognize and treat physical diseases in this patient group. To mitigate these challenges, the “Healthy Heart Tool” was developed, as an easy-to-use Norwegian adaption of the Australian Positive Cardiometabolic Health resource [37] and the British adaption of the Australian resource (the “Lester Tool”) [38, 39].

The aim of the study was hence to investigate whether a broad implementation of the Healthy Heart Tool in health care services improved CVD risk management in persons with SMI, by comparing differences in risk factor awareness at baseline and after 12 months of implementation and to investigate whether there was an improvement of cardiometabolic risk factors among the participants.

## Methods

### Design/ setting

This was a multi-center observational study including persons with SMI admitted to adult mental health care (hospitals or outpatient services) in six Norwegian health trusts between December 2016 and April 2018. The hospitals included are Oslo University Hospital, Diakonhjemmet Hospital, University Hospital of North of Norway, Helse Bergen Hospital Trust, Vestfold Hospital Trust and St Olav’s Hospital Trust. The study is part of the larger Healthy Heart Study, a multi-center study organized by the Norwegian Research Network in Severe Mental Illness (NORSMI).

### Study population

People aged 18–90 years with a clinical ICD-10 diagnosis of schizophrenia spectrum disorder (F20-29), the affective spectrum disorders F30, F31, F32.3 and F33.3 or current use of antipsychotic medication were eligible for inclusion. Exclusion criterion was inability to give informed consent.

Information about the project was given to eligible candidates by clinical personnel working at inpatient and outpatient specialist mental health care, both in one-to-one sessions and in information meetings. The eligible individuals were then referred to research staff at each site if they wanted to participate. The questionnaires applied were the same at all sites, and a local project coordinator was responsible for standardizing the data acquisition process in accordance with the protocol. All participants signed a written informed consent before participating in the study, and it was clearly emphasized that non-participation would not lead to negative consequences.

### Participants

Two hundred and seventy individuals were included in the study, 151 men (55.9%) and 119 women (44.1%). Sixteen per cent of the study sample had bipolar disorder and 3% had a diagnosis of substance disorder. Seventy-seven per cent of men and 70% of women had schizophrenia disorder. Average age of first treatment was 29.3 years for men and 30.9 years for women.

### Implementation of the Healthy Heart Tool

The Healthy Heart Tool is an algorithm targeted towards health care workers and the health care system in general, not the individual patient. It is based on Australian Positive Cardiometabolic Health resource [37] and the British Lester Tool [38]; translated and adapted to be compatible with the Norwegian health care system and Norwegian guidelines for treatment of patients with SMI and/or substance abuse. The purpose of the tool is to provide guidance to health care workers on how to screen and intervene on CVD risk in persons with SMI, with an easy-to-follow algorithm widely distributed in the health services. The tool highlights threshold-defined “Risk Zones,” type of intervention and treatment aims for six main areas: daily smoking, sedentary lifestyle and poor diet, overweight, blood pressure, glucose regulation and blood lipids. The tool has not been used in a Norwegian setting before the implementation started.

The implementation of the Health Heart Tool was based on a general distribution throughout each participating unit by mail, handouts and meetings. National seminars and local meetings with health care workers in the participating hospitals were organised, to emphasis the importance of physical activity, dietary advice, and information on smoking cessation. The goal was to enhance general awareness based of the tool, therefore there was no individualized training. Implementation started immediately after baseline at all participating sites, with a follow-up of 12 months. Due to different time points for baseline at the participating sites, the local intervention varied both regarding activity and duration. The tool was also included in the national guidelines for CVD risk in persons with SMI and substance abuse during the study period [39]. The result of the joint implementation was a broad, national distribution of the Healthy Heart Tool and hence an increased focus on medication, activities and lifestyle advice, demanding a higher level of attention and responsibility for CVD health in patients treated within psychiatric health care.

### Assessments

The research staff at all participating sites interviewed patients according to a semi-structured questionnaire. Information was validated with patient record information. If data were deficient due to participants imprecise knowledge or memory, it was supplemented with information from patient records. Sociodemographic information included age and gender, urban/rural living, ethnicity, education, occupation, housing and marital/civil status. Global symptoms and psychosocial functioning were measured by the Global Assessment of Functioning Scale (GAF), and the scores were split into scales of symptoms (GAF-S) and functioning (GAF-F) to improve psychometric properties [40]. Life satisfaction was assessed with the life satisfaction questionnaire from HUNT 3-Q1 [41].

Information on cardiometabolic risk at baseline and 12 months prior to inclusion was collected from questionnaires and medical records. Daily smoking was recorded as number of cigarettes per day and level of physical activity was registered according to frequency, duration and intensity. Sedentary behavior was defined as one session of physical activity or less per week [42]. Diet was registered as “healthy”, “moderately healthy” and “very unhealthy,” assessment were conducted by research staff based on diets composition and national dietary guidelines [43].Use of illegal substances the past six months was registered. Somatic comorbidity including cardiovascular disease, hypertension, diabetes mellitus and lipid abnormalities with year of detection collected from questionnaires and patient records, as well as information about current use of antipsychotic drugs and other medication.

Somatic measurements at baseline and after 12 months was conducted on each participant and included weight and height (with calculation of Body Mass Index (BMI)), waist circumference, systolic and diastolic blood pressure. Blood samples were taken by the General Practitioner (GP) or at the hospital, and included hemoglobin A1C (HbA1c), non-fasting plasma glucose and non-fasting plasma lipids (high density lipoproteins (HDL) cholesterol, low density lipoproteins (LDL) cholesterol, total cholesterol and triglycerides). Both glucose and lipids were taken non-fasting to assure samples in cases where patients were out of hospital and participation was possible only in out-patient’ settings.

Reception of advice according to the Healthy Heart Tool, such as smoking cessation advice, guided participation in physical activity or dietary guidance according to national guidelines were registered as “received intervention”. Intervention is part of the national guidelines after baseline and its therefore no way to divorce intervention trough guidelines from sole intervention by the tool. It was not assessed at what exact time intervention was given, just if it was given during the last 12 months before baseline inclusion and follow-up. Prescription of blood pressure medication, metformin hydrochloride and statins, and reassessment of psychotropic medication due to side effects were also registered. As for clinical examination we asked at baseline and after 12 months if plasma glucose, HbA1C, blood lipids, body weight, waist circumference and blood pressure has been assessed by either the GP, in specialist health care or both during the last 12 months.

Due to small numbers some categories were merged after sampling based on similarity, for example dietary advice and advice on salt restriction.

Cardiometabolic risk interventions were grouped according to risk factors into (1) clinical examinations performed and (2) preventive measures taken. Interventions performed by specialist health care, GP or both were registered separately in the questionnaire, but were merged in the analyses.

### Analysis and statistical methods

Demographic and clinical characteristics were summarized for all participants in total. Prevalence of risk factors were described as defined in the Healthy Heart Tool.

We calculated binary indicator variables corresponding to the risk factors in the Healthy Heart Tool as, BMI ≥ 30, total cholesterol ≥ 5 and ≥ 7, LDL cholesterol ≥ 4 and HbA1c ≥ 6.5%. Paired two-sample t-tests and McNemar’s tests were used to compare differences in intervention rates between participants with data at both baseline and follow-up in continuous and dichotomous variables respectively.

Our data had a substantial amount of missing data at 12 months for several of the variables. Reasons for this included an inability or unwillingness to show up for follow-up, which in turn may have been due to increasing symptoms, moving or loss of contact with the healthcare system. For that reason, we decided to apply a missing data imputation technique, to avoid running statistical analyses on a small sample size.

Mechanisms for missing data could be due to data missing completely at random (MCAR), missing at random (MAR), or missing not at random (MNAR). While it is practically difficult to verify the missing data mechanism, a common approach is to rule out MCAR and assume MAR [44]. Thus, we presumed our data comply with a MAR assumption and hence chose a missing data imputation method that is valid under MAR; an Expectation Maximization (EM) algorithm. EM first estimates the expected values of the missing data points, based on the available data, and then maximizes the log-likelihood of the missing variable distribution. These two steps are repeated until convergence of the log-likelihood, i.e., when there are only minor differences in the estimated likelihood between the subsequent steps. Using the EM method in SPSS, the following variables were imputed: BMI, LDL, HDL, cholesterol, HbA1c, waist in cm, smoking yes/no, gender. The resultant complete dataset was then imported into R as the basis for the generalized linear mixed models performed.

Although we did not apply any formal sensitivity analyses to rule out other possible missing data patterns (such as MNAR), we have made a crude comparison of datasets imputed by EM and by multiple imputation method in SPSS. In our opinion, the imputed values did not vary to such an extent as to radically affect the results of the statistical models.

To analyse the effect of implementing the Healthy Heart Tool, we used logistic general linear mixed model (GLMM). For the binary dependent variable “receiving dietary intervention” (yes/no) we fitted two different models. The first model included the independent variables: Binary time (effect of the Healthy Heart Tool at follow-up), gender, BMI, total cholesterol, LDL cholesterol and HbA1c as predictors. The second model only included binary time, gender and BMI as predictors.

For the second binary dependent variable “reassessment of medication due to side effects” intervention we also used a logistic GLMM regression model. The first model only included the independent variable binary time (effect of Healthy Heart Tool at follow-up), and the second model included binary time and gender.

For all models, the identifier for each individual was used as a random factor to account for dependencies due to repeated measurements in the data between baseline and follow-up.

We used the R package LME4 in R (version 4.1.3) to calculate the GLMM regression models. The option “nAGQ” in the “glmer” function was set to 11 to use the adaptive Gauss-Hermite quadrature instead of the default Laplace approximation, since the adaptive Gauss-Hermite quadrature is considered more accurate [45].

## Results

Demographic and clinical description of the sample at baseline is presented in Table 1.

**Table 1:** Demographic and clinical characteristics including cardiometabolic risk factors at baseline. Total responders (n) indicate numbers of participants responding to this item

	Total	Total responders (*n*)
Sex female/male	119/151	
Age [mean (range, total years)]	44.7 (18–87)	270
Higher education (more than 12 years) [n, (%)]	122 (46.9)	260
Married or cohabiting [n, (%)]	53 (20)	266
Privat residence [n, (%)]	184 (69)	266
Duration of hospitalization (mean, [range min-max months])	18.5 (0.10–240)	207
Age of first treatment (mean ± SD)	26.5 (12.4)	237
Duration of treatment (mean, [range min-max months])	18.5(1–57)	237
*Diagnoses*		
Substance induced psychosis [n, (%)]	7 (2.7)	263
Schizophrenia spectrum^a^ [n, (%)]	200 (76)	263
Affective spectrum disorder ^b^ [n, (%)]	43 (16)	263
Other^c^ [n, (%)]	13 (4.9)	263
GAF-S^d^ (mean, [range min-max])	49.6 (20–99)	231
GAF-F^e^ (mean, [range min-max])	49 (20–90)	232
Life satisfaction^f^ (mean, [range min-max])	3.6 (1–7)	255
*Comorbidity*		
Cardiovascular disease [n, (%)]	79 (29.5)	268
Other [n, (%)]	189 (70.5)	268
Use of illegal drugs past 6 months [n, (%)]	38 (14.9)	255
Total cholesterol ≥ 5 [n, (%)]	141 (54.4)	259
Total cholesterol ≥ 7 [n, (%)]	9 (3.5)	259
LDL cholesterol ≥ 4 [n, (%)]	57 (22)	259
Body mass index ≥ 30 [n, (%)]	101 (39.3)	257
HbA1c ≥ 6.5 [n, (%)]	15 (6.0)	251
Daily smoking (mean ± SD)	102 (38.8)	263

^a^ ICD 10 F 20-F29

^b^ ICD 10: F30, F31, F32.3 and F33

^c^ ICD 10: F06.31, F06.2, F07.0, F42.2, F41.8, F43.1, F44.8, F84.5, F99, R45.8

^d^ Global Assessment of Symptoms (split version)

^e^ Global Assessment of Functioning (split version)

^f^ The life satisfaction questionnaire from HUNT [41]

There were 151 men and 119 women participating in this study, mean age was 44.7 years. Schizophrenia spectrum disorders were the main diagnoses, and duration of hospitalisation ranged from 0.10 to 240 months with a mean of 18.5. Almost 50% of participants had higher education, 12 years and more and almost 70% lived in private housing. For 7 participants, the diagnose were not conclusive.

Table 1 also shows the distribution of cardiometabolic risk factors among the participants. Almost 30% had a cardiovascular disease, including hypertension. Half of the participants had total cholesterol ≥ 5 mmol/l, only a few had total serum cholesterol ≥ 7mmol/l. Almost 40% of the participants were defined as overweight with BMI ≥ 30, and almost 40% were daily smokers. As shown in Table 2, for most of the participants (90%) clinical examination with measurements of risk factors were performed, with no significant difference between baseline and 12 months follow-up.

**Table 2:** Performed clinical examination, cardiovascular risk factors and measures at baseline and 12 months follow-up^a^. Total responders (n) indicate numbers of participants responding to this item

Clinical examination	Baseline [*n*(%)]	Total responders ^b^ (*n*)		12 months [*n*(%)]	Total responders ^b^ (*n*)		*P*-test
Plasmaglucose	207 (94.5)	219		124 (92.5)	134		0.344
Hemoglobin A1c	206 (93.6)	220		130 (95.6)	136		1.000
Lipids	219 (96.1)	228		131 (94.2)	139		0.549
Body weight	210 (92.5)	227		150 (96.2)	156		1.000
Waist circumference	139 (83.7)	166		126 (88.7)	142		0.267
Blood pressure	204 (94.0)	217		149 (97.4)	153		1.000
**Cardiometabolic risk factors**							
Total cholesterol ≥ 5	141 (54.4)	259		74 (46.3)	160		0.016
Total cholesterol ≥ 7	9 (3.5)	259		5 (3.1)	160		1.000
LDL cholesterol ≥ 4	57 (22.0)	259		24 (15.6)	154		0.169
Body mass index ≥ 30	101 (39.2)	257		69 (39.2)	176		1.000
Hemoglobin A1c ≥ 6.5	15 (6)	251		19 (11.8)	161		0.012
Daily smoking	102 (38.8)	263		62 (34.3)	181		0.424
Body weight (mean ± SD)	86,2 (± 22.8)	262		86,5 (± 22.2)	182		0.088
Waist circumference (mean ± SD)	100,8 (± 18.3)	239		101,2 (± 18.2)	182		0.198
**Measures**							
Smoking cessation advice/program	52 (51.0)	103		49 (60)	82		0.180
Motivational intervention organized activity	173 (86.5)	200		139 (88.5)	157		0.503
Dietary advice and guidance/advice on salt reduction	114 (75.5)	151		112 (84.8)	132		0.035
Referal to a dietary specialist	16 (18.8)	85		12 (16.9)	71		1.000
Antihypertensiv medication (current prescription)	30 (34.9)	86		31 (41.3)	75		1.000
Metformin (current prescription)	16 (18.6)	86		14 (20.6)	68		1.000
Statins (current prescription)	21 (25.3)	83		21 (30.4)	69		0.250
Reassassment of medication due to side effects	55 (50.9)	108		55 (57.9)	95		0.035

^a^ N describes numbers of persons receiving examination or measures, and p-value indicates the difference between baseline and 12 months follow-up

^b^ Total numbers of answers on this item

For cardiometabolic risk factors, there was a significant reduction in percentage of participants with high levels of total serum cholesterol ≥ 5 mmol/l, a non-significant change for LDL cholesterol ≥ 4 mmol/l and no change for BMI ≥ 30. There was a small, but non-significant reduction in the percentage who were daily smokers. We also found a significantly higher percentage of participants with HbA1c ≥ 6.5 mmol/ml at 12 months follow-up compared to baseline. Men had more comorbid CVD than women (31.8% vs. 26.1%, respectively, results not shown in Table 2).

Table 2 shows a significantly higher proportion of persons receiving dietary advice and/or salt restriction advice at 12 months follow-up than at baseline and significantly higher proportion of persons experiencing changes in prescribed medication due to side effects at 12 months follow-up.

To investigate if rates of intervention from health care professionals had changed from baseline to 12 months follow-up we conducted a GLMM analysis as displayed in Table 3. Model 1 displays several risk factors, but the model did not converge, possibly because of similarity between the indicator variables. When we controlled for potential risk factors, we found a significant increase in persons receiving dietary advice and/or salt restriction from baseline to 12 months, with an odds ratio (OR) of 3.6. BMI ≥ 30 was significantly associated with receiving such advice, with OR = 7.1 after controlling for relevant risk factors. For persons having HbA1c > 6.5 there was a non-significant OR = 9.6 for receiving dietary advice and/or salt restriction.

**Table 3:** Logistic regression models using dietary intervention as dependent variable^a^. Model # 1 full Healthy Heart Tool model^b^

Risk factors	Odds ratio	Std. Error	95% CI		*P*- value
Time	3.6	0.6	(1.03–12.79)		0.045
Female sex	1.7	0.7	(0.39–7.07)		0.496
Body mass index ≥ 30	7.1	0.9	(1.19–42.03)		0.031
Total cholesterol ≥ 7	0.3	2.2	(0.003–22.50)		0.572
LDL cholesterol ≥ 4	1.3	1.0	(0.19–8.14)		0.808
HbA1c ≥ 6.5	9.6	2.1	(0.15-601.02)		0.284
Time	3.6		(1.04–12.54)		0.043
Female sex	1.6		(0.39–6.89)		0.501
BMI ≥ 30	8.9		(1.42–55.77)		0.019

^a^ General linear mixed model (GLMM) applied

^b^ from baseline to 12 months follow-up

After controlling only for BMI ≥ 30 and gender in model 2 there was an OR of 8.9 for receiving dietary advice and/or advice on salt reduction.

The McNemar’s test showed that a significantly higher proportion had received reassessment of medication due to side effects after 12 months follow-up. However, the logistic GLMM regression analysis displayed in Table 4 shows a non-significant OR of 2.6 for change from baseline to 12 months. Controlling for gender did not change this result.

**Fig. 1:**
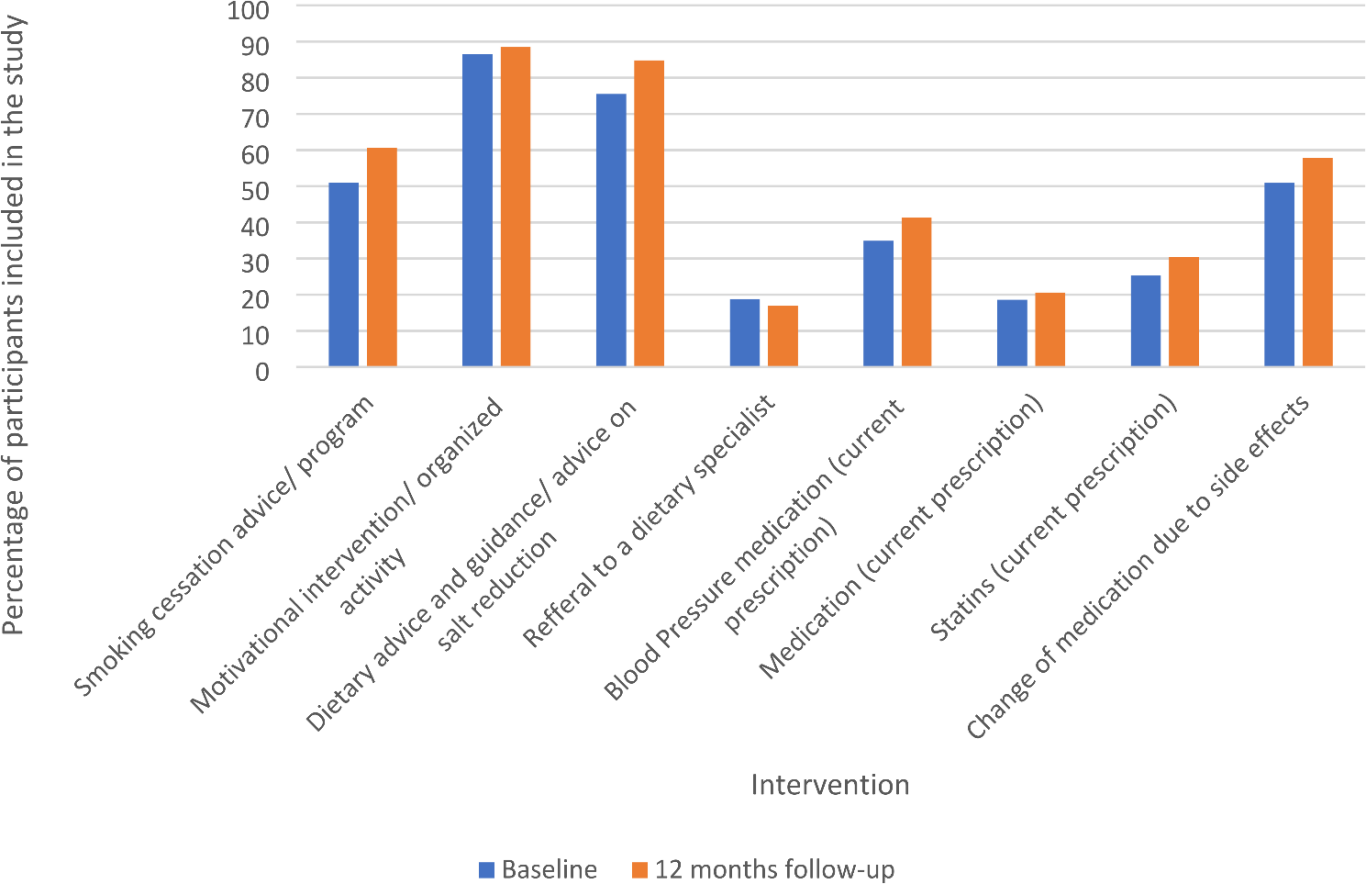
Intervention by health care professionals from baseline to 12 months follow-up

Figure 1 shows the change in intervention rate from health care professionals from baseline to 12 months follow-up. The figure visualizes a possible trend of increased attention towards cardiovascular intervention in patients with SMI between baseline and 12 months follow-up, but this must be interpreted with caution.

**Table 4:** Logistic regression models using reassessment of medication due to side effects as dependent variable^a^. Model # 1 Reassessment of medication due to side effect^b^, Model #2

Risk factors	Odds ratio	Std. Error	95% CI	P-value
Time	2.6	0.6	(0.83–7.50)	0.102
Time	2.4	0.7	(0.79–7.03)	0.124
Female sex	1.6	0.7	(0.58–9.77)	0.224

^a^ General linear mixed model (GLMM) applied

^b^ from baseline to 12 months follow-up

## Discussion

The main findings in this study are that a significantly higher proportion of patients at risk received dietary advice and/or advice on salt restriction one year after implementation of the Healthy Heart Tool. The differences remained significant after controlling for gender and obesity. Obesity was significantly associated with receiving such advice, whereas abnormal levels of total cholesterol, LDL cholesterol and HbA1c did not affect the difference in intervention measures between baseline and follow-up.

At the patient level, we found that nine out of ten persons underwent measurement of their body weight by their GP or in specialized health care both at baseline and follow-up, and a similar proportion of participant had been screened for CVD risk with measurement of lipids, blood pressure, HbA1c, weight and/or blood pressure at both time points. There was a significant reduction (*p* = 0,016) in proportion of participants with high level of total serum cholesterol ≥ 5 mmol/l (54,4% vs. 46,3%).

Our findings are in line with results observed after implementation of the Lester Tool in UK, where more in-patients received screening after implementation (from 46% at baseline to 83% at follow-up). Also intervention rates increased from 79 to 94% for participants shown to be in need of treatment as a result of screening [46]. In our study there was a nominally increase in 7 of 8 domains of interventions from baseline to follow-up, but apart from dietary advice and/or advice on salt restriction, there were few significant changes. This may reflect that the implementation of new routines or guidelines is a complex undertaking. There is a well-known lack of adherence to new guidelines both at the individual level and at the organizational and system-based level [47]. The Healthy Heart Tool is, however, an easy-to-use algorithm based on already well-known national guidelines, and implementation should therefore be less demanding. “Unlearning” former knowledge may also impact practitioners’ ability to change clinical practice and to include a new algorithm in their daily work [48]. According to the Healthy Heart Tool, screening is the first step and should be followed by intervention, such as customized medication or health advice. Blood samples and measurement of blood pressure, body weight and waist circumference are available and simple examination methods, whereas interventions such as motivational interview, referrals and medication demand continuous follow-up and several consultations. In many cases, blood samples taken in in-patient units and post-discharge follow-up (either from GP or from the hospital), are needed.

The high number of persons with obesity found in our study is in line with previous literature, showing that people with SMI are three times more likely to have obesity compared to the general population [49]. Before the implementation of the Healthy Heart Tool in Norway in 2018, almost 90% of persons included were defined as being in the Risk Zone and in need of interventions [50]. Half of the persons were screened according to all cardiometabolic risk variables. More than 50% of the persons were obese, and receiving intervention dietary advice and guidance were associated with obesity and high waist circumference.

The combination of diabetes, high cholesterol, smoking and obesity leads to greater risk of CVD and might call for treatment with statins to reduce CVD risk [51]. In the present study, the share of persons with current prescription of statins increased numerically during follow-up, but this was not significant. A lower prescription rate of statins has been shown for persons at increased CVD risk with SMI [31], whereas a recent cohort study from UK describes higher levels of initiation of statins for persons aged 30–59 years with SMI [52].Statin treatment for persons at younger age has also been discussed for the general population [53]. We observed a slight, numerically non-significant increase in current prescription of metformin hydrochloride, possibly due to the observed increase in persons with HbA1c ≥ 6,5 between baseline and follow-up. About half of the participants had received smoking cessation advice from their GP or in specialized health care, with no significant difference between baseline and follow-up.

Overweight and obesity clearly indicate a need for medical intervention, and advice on diet for weight reduction is easy and non-invasive. As obesity is a visible risk factor, the threshold for dietary advice is possibly low, but the question remains whether dietary advice is enough in the long term. The high number of persons receiving such dietary advice and/or advice on salt reduction stands in contrast to the low numbers of persons referred to a dietary specialist, which may indicate a lack of knowledge on where to refer these persons, or a high threshold for referral to specialized health care.

### Strengths and limitations

The main strength of the current study is the pragmatic design in a real-world setting with a broad inclusion of high-risk persons with SMI assessed for CVD risk factors and health care measures before and after implementation. The strength of the Healthy Heart algorithm is the overall presentation of cardiometabolic risk factors that may improve adherence in daily practice in both specialised and general practice.

The wide inclusion in the study gives the opportunity to study a representative selection of persons with SMI, and thereby a wider spectrum of CVD-risk factors with possible reduction of selection bias.

There are, however, several limitations.

Because there was no direct linkage between individual participants and the use of the healthy Heart Tool, our results reflect a general increased attention towards cardiometabolic health in persons with SMI rather than improved individualised follow-up. There was no control group in the study, due to the widespread national distribution, and robust conclusions concerning significant effects of the implementation are therefore not possible to make.

Further, because we do not have the direct point of time in which intervention was given after baseline, the time span between intervention and follow-up might be too short for measurable changes in metabolic features.

Moreover, the high number of missing participants at follow-up makes the estimates of the difference between baseline and follow-up uncertain. The short timespan between baseline and follow-up is also a limitation, in particular because tentative changes of habits and clinical practice takes time [48, 54]. Furthermore, persons with BMI > = 30 are not divided into subgroups, due to the limited sample size. We therefore do not know whether persons have gone through a transition and become more, or less overweight. We do not have information on persons who did not participate, but the characteristics of those included in the study are however comparable to other samples regarding sociodemographic information, diagnoses or level of functioning [55]. Recall bias concerning information in the questionnaires is also possible, albeit limited by validation of information from medical records. Further, it may be that the participants with higher motivation and interest and/or a higher degree of compliance met at the follow-up, which may have led to an exaggerated estimation of change.

Due to the high CVD morbidity and mortality in persons with SMI, it is important to identify risk factors and possible interventions at an early stage. How to increase clinicians’ awareness is, however, complex. To move from what one *knows* to what one *does* is difficult [56]. To change practice, a shared vision that includes knowledge about why adherence to the algorithm will make a difference, is needed.

All in all, we cannot interpret changes in risk factors as direct results of the Healthy Heart Tool. The results from such a pragmatic and broad implementation may still reflect a higher level of attention towards cardiometabolic risk in persons with SMI. Also, this is often the way changes are imposed on the health care services. To study possible implementation effects hence gives us some indications of both feasibility and areas for further research.

## Conclusion

We found a significantly higher proportion of patients with SMI that received dietary advice and/or advice on salt reduction after 12 months of nationwide implementation of the Healthy Heart Tool. A reduction in CVD risk factors such as cholesterol level was observed. The findings are a promising indication that the broad, national implementation of the Healthy Heart Tool have led to increased attention among health care professionals towards cardiovascular screening, monitoring of CVD risk, and if necessary, interventions in persons with SMI, but further research is needed to investigate direct effects.

## Data Availability

The dataset used and analysed during the current study are available from the corresponding author on reasonable request.

## Abbreviations

BMI: Body mass index
SMI: Severe mental illness
OR: Odds ratio
CI: Confidence interval
CVD: Cardiovascular disease
NICE: National Institute for Health and Care Excellence
NORSMI: Norwegian Research Network in Severe Mental Illness
ICD-10: International Classification of Diseases, 10th revision
GAF-F: Global Assessment of Functioning-function aspect
GAF-S: Global Assessment of Functioning-symptom aspect
GP: General practitioner
HbA1c: Hemoglobin A1C
HDL: High density lipoproteins
LDL: Low density lipoproteins
MCAR: Missing completely at random
MAR: Missing at random
MNAR: Missing not at random
EM: Expectation Maximization
GLMM: General linear mixed model
